# Preventive effects of basic fibroblast growth factor on vascular restenosis after balloon angioplasty

**DOI:** 10.3892/etm.2014.1562

**Published:** 2014-02-19

**Authors:** FENG RAN, CHANGJIAN LIU, ZHAO LIU, TAO SHANG, MIN ZHOU, TONG QIAO

**Affiliations:** Department of Vascular Surgery, Affiliated Drum Tower Hospital of Nanjing University Medical School, Nanjing, Jiangsu 210008, P.R. China

**Keywords:** basic fibroblast growth factor, angioplasty, vascular endothelium

## Abstract

The aim of the present study was to investigate whether chronic administration of basic fibroblast growth factor (bFGF) following angioplasty in a dog model of atherosclerotic iliac stenosis may restore endothelium function and prevent restenosis (RS). In total, 40 dogs with atherosclerotic stenosis of the right iliac arteries were used in the study. A total of 20 dogs underwent histological examination of the lumen areas prior to (n=10) and immediately following angioplasty (n=10). Intravenous bFGF was administered to 10 dogs (bFGF group) and an additional 10 dogs received vehicle injection (control group). Animals in the two groups were sacrificed 42 days following surgery for *in vitro* analysis of vascular reactivity and morphometric assessment of the histological cross-sectional areas. The bFGF group exhibited significantly greater maximal endothelium-dependent acetylcholine-induced relaxation (E_max_, 43±9%) when compared with the control group (E_max_, 8±6%; P<0.05). In addition, the maximal endothelium-independent response of the bFGF group to sodium nitroprusside (E_max_, 90±2%) was greater than that of the control group (E_max_, 60±2%; P<0.05). Six weeks following angioplasty, the lumen area in the bFGF group (2.01±0.78 mm^2^) was greater compared with the control group (1.0±0.10%). The lumen area decreased by 58% between immediately after angioplasty and the control group six weeks following angioplasty. Therefore, the results of the present study indicated that administration of bFGF may not only restore endothelium-dependent and -independent relaxation, but also prevent RS in dogs that have undergone angioplasty.

## Introduction

Angioplasty is an effective method to arteriosclerosis. Restenosis (RS) is a common adverse event of angioplasty, which is a complicated formation. In the present study, the preventive effects of basic fibroblast growth factor (bFGF) on RS were observed. In addition, the effects of bFGF administration on endothelium-dependent and -independent vasorelaxation were analyzed during angiopoiesis.

## Materials and methods

### Animals

A total of 40 male experimental dogs (weight, 12.5 kg) were fed a specific diet each morning (cholesterol, 5 g, Sigma-Aldrich, St. Louis, MO, USA; fat, 15 g, Sigma-Aldrich; egg yolk, 50 g; common mixed feed, 500 g). After 3 weeks, the animals were administered 0.5 mg/kg chloral hydrate (Medical Isotopes, Inc., Pelham, NH, USA) diluted in 10% alcohol (Anyang General Chemical Co., Ltd., Anyang, China) via an intravenous drip. Following endotracheal intubation, a breathing apparatus was connected to perform ventilation. Animals were anesthetized with ketamine hydrochloride (25 μg/kg^−1^/min, Betaphase Chemicalz, New York, NY, USA) and flaccidity was sustained with suxamethonium chloride (25 μg/kg^−1^/min, Chemical Point UG, Deisenhofen, Germany). Lidocaine (Changzhou Longterm Biotechnology Co., Ltd., Changzhou, China) was used to lower the heart rate and avoid arrhythmia. The femoral artery and vein were dissected, arterial pressure was determined and fluid replacement was maintained through the femoral vein. Following right iliac endarterectomy (size, 30 mm; Takumi balloon, Schneider Corporation, Indianapolis, IN, USA), the animals were fed a hypercholesterolemic diet for 15 weeks. The study was performed in strict accordance with the recommendations in the Guide for the Care and Use of Laboratory Animals of the National Institute of Health. The animal use protocol was reviewed and approved by the Institutional Animal Care and Use Committee of the Affiliated Drum Tower Hospital of Nanjing University Medical School, (Nanjing, China).

### Level of atherosclerotic stenosis

In total, 10 dogs were used to examine the level of atherosclerotic stenosis in the iliac arteries. In order to maintain the size, the iliac arteries were perfused with 4% polyoxymethylene (Ticona GmbH, Frankfurt, Germany) diluted in phosphate-buffered saline (Lorad Chemical Corporation, Petersburg, VA, USA) for 40 min with a constant pressure of 25.0 kPa through a duct from the right carotid artery to the abdominal aorta. Subsequently, the samples were further fixed with 4% polyoxymethylene, embedded in paraffin and cut into sections. The slides were stained with hematoxylin and eosin (Dudley Chemical Corp, Lakewood, NJ, USA). Images were captured to detect the cross-sectional areas of the iliac arteries on both sides and to identify atherosclerotic stenosis using a digital image analyzer (Quantiment-520; Leica, Cambridge, UK).

### Balloon dilation

A balloon (size, 30 mm; Takumi) was placed at the stenosis region via the femoral artery and inflated to 706.6 kPa for 3 min. This was conducted three times consecutively at 1 min intervals and then the balloon was removed. Following angioplasty, 10 experimental dogs were randomly selected from 30 dogs. These dogs were used to analyze the instant changes of the lumen areas following angiopoiesis. The remaining 20 dogs were randomly divided into the control and bFGF groups (n=10 per group). The dogs in the bFGF group were administered 5 μg recombinant bFGF (Sigma-Aldrich, St. Louis, MO, USA) dissolved in 1 ml albumin (0.5%, w/v) via an intravenous drip on days 1, 4, 7, 10 and 14 following angioplasty. The experimental dogs in the control group were treated with 1 ml albumin (0.5%, w/v). Dogs in the two groups were sacrificed 42 days after angioplasty to analyze the vascular reactivity and histology *in vitro.*

### Histology

A total of 10 dogs were divided into two groups (n=5) and the samples were harvested, fixed, stained and detected as aforementioned.

### In vitro vascular reactivity

Six weeks following angioplasty, iliac arteries on both sides (angiopoietic and non-angiopoietic) were harvested and divided into groups (n=5 in each group). The arteries were cut into vascular circles, 10-mm in length. The vascular circles were dipped in 40 ml K-H solution (pH 7.4; Sigma-Aldrich) and incubated in conditions of 95% O_2_ and 5% CO_2_ at 37°C. Vascular circles were connected with the pressure translator, which was connected to a physiological recorder (VM-180G, Optical-Electric Co., Hyogo, Japan). Prior to administration, the vascular circles were stabilized for 60 min at an optimal resting tension of 4 kPa. All the arterial circles were pretreated with phenylephrine (Phe; 1×10^−9^–3×10^−5^ mol/l; Pechiney, Stamford, CT, USA), which resulted in stenosis. Following stabilization of the Phe-induced contractile response, all arterial circles were treated with endothelium-dependent and -independent vasodilator. With the administration of acetylcholine (ACh; 1×10^−8^–3×10^−5^ mol/l; Angene International Limited, Hong Kong, China), the arterial circles were rinsed and stabilized at resting tension when the maximal relaxation occurred. The smooth muscle relaxant, sodium nitroprusside (SNP; 1×10^−9^–3×10^−5^ mol/l; Angene International Limited), was added to the prestenotic arterial circles treated with Phe (3×10^−5^ mol/l).

### Statistical analysis

Results are expressed as the mean ± SD and data were analyzed using the t-test. P<0.05 was considered to indicate a statistically significant difference. ACh- and SNP-induced relaxation was calculated as a percentage of the contraction to Phe. E_max_ was the maximal vascular reactivity and EC_50_ was 50% of the maximal reaction concentration.

## Results

### Iliac artery area

**Figure 1:**
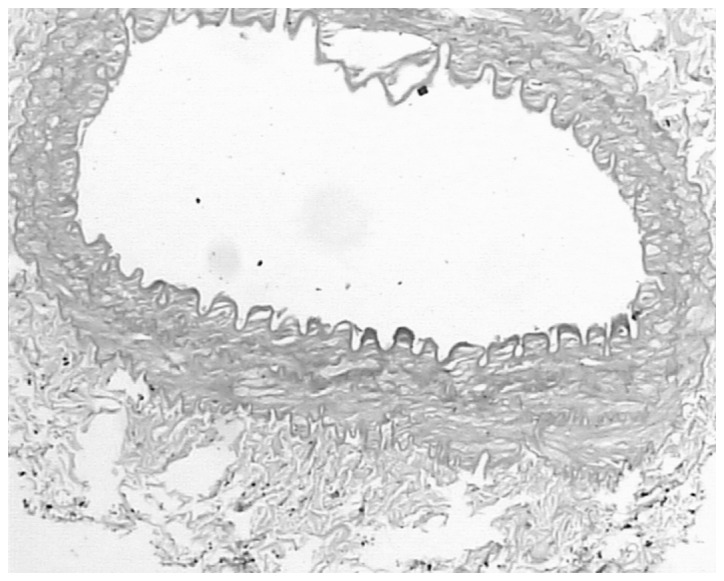
Hematoxylin and eosin staining of the bFGF group following angioplasty. bFGF, basic fibroblast growth factor.

**Figure 2:**
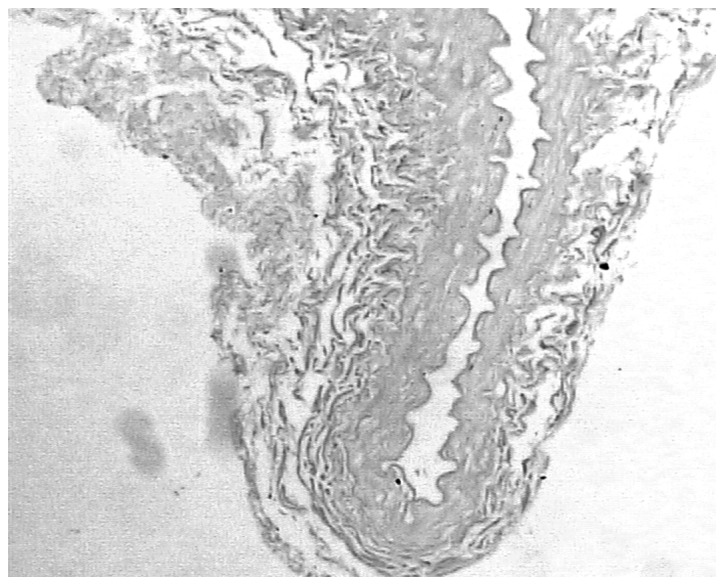
Hematoxylin and eosin staining of the control group following angioplasty.

Following angioplasty, cross-sectional areas of the intimas following endarterectomy on the right side were 60.5% smaller compared with the intimas that had not undergone endarterectomy (0.89±0.79 vs. 2.88±0.89 mm^2^; P<0.05). Instant cross-sectional areas on the right side after angioplasty had an average area of 2.10±0.34 mm^2^, which was significantly greater compared with the area before angioplasty (0.89±0.79 mm^2^; P<0.05). Changes in cross-sectional areas of the right iliac arteries 6 weeks after angioplasty are indicated in Table I and Figs. 1 and 2.

### In vitro vascular reactivity

The contractile response resulting from Phe administration exhibited no significant differences between the angiopoietic and non-angiopoietic sides of the iliac arterial circles in the control and bFGF groups. In addition, there was no significant difference in the endothelium-independent SNP-induced vasorelaxation response of the non-angiopoietic iliac arterial circles between the control and bFGF groups (E_max_, 93±5.5 vs. 91±4.5%; P>0.05; EC_50_, 8.9±4.3 vs. 8.8±4.1×10^−7^ mol/l; P>0.05). Additionally, there was no significant difference in the endothelium-dependent Ach-induced vasorelaxation (E_max_, 53±7.5 vs. 55±6.7%; P>0.05; EC_50_, 7.8±2.1×10^−7^ mol/l vs. 7.5±2.1×10^−7^ mol/l; P>0.05). Six weeks after angioplasty in the right iliac arteries, the maximal endothelium-dependent Ach-induced relaxation of the bFGF-treated animals (E_max_, 43±8.5%) was significantly greater compared with the control group (E_max_, 11±9%; P<0.05). Furthermore, E_max_ and EC_50_ values of the right side after endarterectomy recovered to the level of the opposite side without endarterectomy in the bFGF group (E_max_, 50±8 vs. 45±7%; P>0.05; EC_50_, 8.1±2.0 vs. 7.8±2.1×10^−7^ mol/l; P>0.05). Following angioplasty, the maximal endothelium-independent SNP-induced relaxation response of the angiopoietic sides was markedly weaker compared with the non-angiopoietic sides of the iliac arterial circles in the bFGF group (90±2 vs. 93±5.5%; P<0.05) and the control group (60±2 vs. 91±4.5%; P<0.05). However, the E_max_ of the bFGF group was significantly greater compared with the control group (P<0.05).

## Discussion

Following angioplasty, vascular injury, which is analogous to generalized wound healing, may be differentiated into three implicated vascular injury repair stages (1). Firstly, early thrombus formation where the elastic components of the vessel wall are dragged and torn during angioplasty, resulting in intimal and deep dissection extending into the medial and adventitial layers. Dissection planes with endothelial stripping induce exposure of components under the intima, platelet adhesion, thrombus formation, acute inflammatory response and the infiltration of neutrophils, monocytes, macrophages and T lymphocytes (2–4). Secondly, there is the cell granulation stage where relevant cells migrate into the vascular injury region. Cells staining for HHF-35 are present, filling in the original dissection planes and in certain arteries, areas of organized thrombus with interdigitated smooth muscle cells (SMCs) are observed (5,6). Collagenous and elastic fibers are few and foam cells are generally observed (2). Following activation with growth factors, including bFGF and platelet derived growth factor, SMCs metastasize to the injury region, proliferate and secrete extracellular matrix (7–9). Finally, there is the vascular remodeling stage where intimal SMCs are unable to proliferate further. SMCs and adventitial fibroblasts secrete extracellular matrix (10,11). An increased density of intimal-medial fibrosis is observed. Collagen proteins constitute 50% of components in the vessel wall and play an important role in vascular RS. In addition, the glycosaminoglycan, hyaluronan, is also a major component of the vessel wall (12).

bFGF is a single-stranded polypeptide (16 ku) that exhibits mitogenic activity and stimulates angiogenesis (13). In the present study, administration of bFGF increased endothelium-dependent vasorelaxation and also restored endothelium-independent vasorelaxation, further indicating that bFGF may be effective in treating experimental vascular injuries. Whether bFGF results in neointima pachynsis and/or proliferation of medial smooth muscle depends on the dose of bFGF. The aforementioned conditions may be observed following administration of a large dose of bFGF, whereas low dose bFGF is beneficial to endothelial cells, indicating that bFGF is double-edged and highly-efficient as a selective standard. Therefore, low-dose bFGF was applied in this study. Administration of bFGF not only restored the functions of endothelial cells, but also improved the sensitivity of vessel SMCs to endothelium-derived relaxing factor, also known as nitrogen monoxidum (14–17). In the present study, there was no significant difference in the sensitivity of angiopoietic iliac arteries to SNP (EC_50_) between the bFGF and control groups. bFGF upregulates the expression of vascular endothelial growth factor and promotes the function of vasorelaxation to produce a synergistic effect of hypoxia (18,19). Thus, the mechanisms of bFGF in improving endothelium-dependent and -independent vasorelaxation require further study. Six weeks after angioplasty, the cross-sectional areas of the control group dramatically decreased, whereas those of the bFGF group showed no change, indicating that bFGF is critical in preventing the formation of RS following angiopoiesis. Therefore, it is possible that the mechanisms behind restoring endothelial cell function and improving vasorelaxation may be associated with the administration of bFGF (20). In addition, there was no change in the cross-sectional areas of the intima without denudation, indicating that normal endothelial cells are essential for preventing atherosclerotic stenosis.

## Figures and Tables

**Table I:** Changes in the cross-sectional areas of the right iliac arteries 6 weeks after angioplasty in the two groups (mm^2^).

Group	Angiopoietic side	Non-angiopoietic side
bFGF	2.01±0.78^a^	2.89±0.78^b^
Control	1.0±0.11	2.68±0.88

a P<0.05 and

b P>0.05, vs. control group.

Values are expressed as mean ± SD. bFGF, basic fibroblast growth factor.

## References

[1] Kibos A, Campeanu A, Tintoiu I (2007). Pathophysiology of coronary artery in-stent restenosis. Acute Card Care.

[2] Zargham R (2008). Preventing restenosis after angioplasty: a multistage approach. Clin Sci (Lond).

[3] Xie C, Guo Y, Zhu T, Zhang J, Ma PX, Chen YE (2012). Yap1 protein regulates vascular smooth muscle cell phenotypic switch by interaction with myocardin. J Biol Chem.

[4] Mulder HJ, Schalij MJ, Kauer B (2001). Pravastatin and endothelium dependent vasomotion after coronary angioplasty: the PREFACE trial. Heart.

[5] Pozo M, Izquierdo MC, de Nicolás R, Egido J, Ortiz A, González-Cabrero J (2009). Gliotoxin inhibits neointimal hyperplasia after vascular injury in rats. J Vasc Res.

[6] Weakley SM, Wang X, Mu H (2011). Ginkgolide A-gold nanoparticles inhibit vascular smooth muscle proliferation and migration *in vitro* and reduce neointimal hyperplasia in a mouse model. J Surg Res.

[7] Segev A, Aviezer D, Safran M, Gross Z, Yayon A (2002). Inhibition of vascular smooth muscle cell proliferation by a novel fibroblast growth factor receptor antagonist. Cardiovasc Res.

[8] Majesky MW, Dong XR, Hoglund V, Daum G, Mahoney WM (2012). The adventitia: a progenitor cell niche for the vessel wall. Cells Tissues Organs.

[9] Song Z, Jin R, Yu S, Nanda A, Granger DN, Li G (2012). Crucial role of CD40 signaling in vascular wall cells in neointimal formation and vascular remodeling after vascular interventions. Arterioscler Thromb Vasc Biol.

[10] Chadjichristos CE, Matter CM, Roth I (2006). Reduced connexin43 expression limits neointima formation after balloon distension injury in hypercholesterolemic mice. Circulation.

[11] Makiyama Y, Toba K, Kato K (2008). Imatinib mesilate inhibits neointimal hyperplasia via growth inhibition of vascular smooth muscle cells in a rat model of balloon injury. Tohoku J Exp Med.

[12] Sadowitz B, Seymour K, Gahtan V, Maier KG (2012). The role of hyaluronic acid in atherosclerosis and intimal hyperplasia. J Surg Res.

[13] Backes A, Seay U, Sedding DG, Tillmanns HH, Braun-Dullaeus RC (2010). Inhibition of matrix deposition: a new strategy for prevention of restenosis after balloon angioplasty. J Cardiovasc Pharmacol.

[14] Cooney R, Hynes SO, Sharif F, Howard L, O’Brien T (2007). Effect of gene delivery of NOS isoforms on intimal hyperplasia and endothelial regeneration after balloon injury. Gene Ther.

[15] Mnjoyan ZH, Doan D, Brandon JL (2008). The critical role of the intrinsic VSMC proliferation and death programs in injury-induced neointimal hyperplasia. Am J Physiol Heart Circ Physiol.

[16] Yao EH, Fukuda N, Ueno T (2009). A pyrrole-imidazole polyamide targeting transforming growth factor-beta1 inhibits restenosis and preserves endothelialization in the injured artery. Cardiovasc Res.

[17] Indolfi C, Torella D, Coppola C (2002). Physical training increases eNOS vascular expression and activity and reduces restenosis after balloon angioplasty or arterial stenting in rats. Circ Res.

[18] Malabanan KP, Kanellakis P, Bobik A, Khachigian LM (2008). Activation transcription factor-4 induced by fibroblast growth factor-2 regulates vascular endothelial growth factor-A transcription in vascular smooth muscle cells and mediates intimal thickening in rat arteries following balloon injury. Circ Res.

[19] Cheng B, Fu X, Sheng Z (2004). Effect of exogenous basic fibroblast growth factor on proliferation and migration of endothelial cells of partial thickness scald in rats. Zhongguo Xiu Fu Chong Jian Wai Ke Za Zhi.

[20] Chang L, Zhang C, Wu YJ, Zhu RZ (2001). Effects of recombinant human basic fibroblast growth factor on restenosis after arterial endothelial injury in rats. Acta Pharmacol Sin.

